# A Rare Case of High-Grade Atrioventricular Block in Granulomatosis With Polyangiitis

**DOI:** 10.7759/cureus.34774

**Published:** 2023-02-08

**Authors:** Shahkar Khan, Taqi A Rizvi, Saran Teja Velaga, Joanne C Ling, Gennifer Makhoul Wahbah, Nnedindu Asogwa, Mustafa Ahmed, James C Lafferty

**Affiliations:** 1 Internal Medicine, Northwell Health/Staten Island University Hospital, Staten Island, USA; 2 Cardiology, Northwell Health/Staten Island University Hospital, Staten Island, USA

**Keywords:** wegener's granulomatosis complications, cardiac involvement in granulomatosis with polyangiitis, ecg monitoring in granulomatosis with polyangiitis, atrioventricular conduction abnormality, granulomatosis with polyangiiitis

## Abstract

Granulomatosis with polyangiitis (GPA) is an autoimmune disease that affects small and medium-sized vessels. It is classically known to present with renal and respiratory tract symptoms. However, the disease can manifest in other organ systems, especially cardiovascular involvement. Though there are multiple reports of cardiac involvement in GPA, it is not commonly evaluated and is often overlooked in patients with GPA.

Heart disease in GPA has a wide range of presentations ranging from subacute and silent to severe abnormalities, which can prove fatal if not identified and treated appropriately. Identifying cardiac involvement early in patients with no apparent signs can help with prevention strategies and follow-up to avoid significant complications. Pericarditis is the most common pathology noted in GPA, followed by cardiomyopathy, coronary artery disease, valvular disease, and conduction abnormality.

In our report, we present a case of GPA in a young male with asymptomatic conduction abnormality of the heart. Although it was silent at the presentation, identifying the initial electrocardiogram (ECG) changes prompted us to admit him to the telemetry floor. Continuous telemetry monitoring helped us identify the progression of the conduction abnormality, which otherwise could have been missed. This led us to correlate to his symptoms which he later developed during his admission course. His symptoms subsided after prompt treatment.

If not identified early, these cardiac abnormalities can delay management, leading to increased disease burden and morbidity. Hence, essential cardiac work with at least ECG and continuous telemetry monitoring is recommended.

## Introduction

Granulomatosis with polyangiitis (GPA), formerly known as Wegener’s granulomatosis, is an autoimmune, systemic necrotizing vasculitis that affects small and medium-sized vessels. It is primarily associated with antineutrophil cytoplasmatic antibodies (c-ANCA) with specificity against proteinase 3 (PR3), but there may be exceptions [1]. It commonly involves the upper and lower respiratory tracts and the renal system. In addition, limited forms commonly involve upper airways. Cardiac involvement is not common but can clinically appear in multiple presentations ranging from subclinical disease to severe abnormalities such as pericarditis, cardiomyopathy, valvular disease, and conduction disorders [2]. In this case report, we discuss a case of GPA with conduction abnormalities and show the importance of monitoring specifically for cardiac involvement in patients with GPA.

## Case presentation

The patient is a 31-year-old male with a past medical history of acute lymphoblastic leukemia as a child in remission and vitiligo. He presented with complaints of generalized weakness for 10 days, associated with myalgia, arthralgia, bilateral eye erythema, and cough. The history went back to six months prior when he started experiencing symptoms of upper respiratory tract disease with reports of intermittent blockage of the nares, followed by ear pain and difficulty hearing. His symptoms were unresponsive to trials of anti-inflammatory and antihistamine medications. The patient also developed bilateral eye erythema, photophobia, pain, and mild weakness in his lower extremities that started a few days before the presentation. His physical examination revealed bilateral eye injection and lacrimal gland hyperplasia; he had a petechial rash on his lower extremities with sensory deficits. In addition, the patient had decreased range of motion at the right knee joint without any apparent signs of synovitis or effusion.

**Figure 1:**
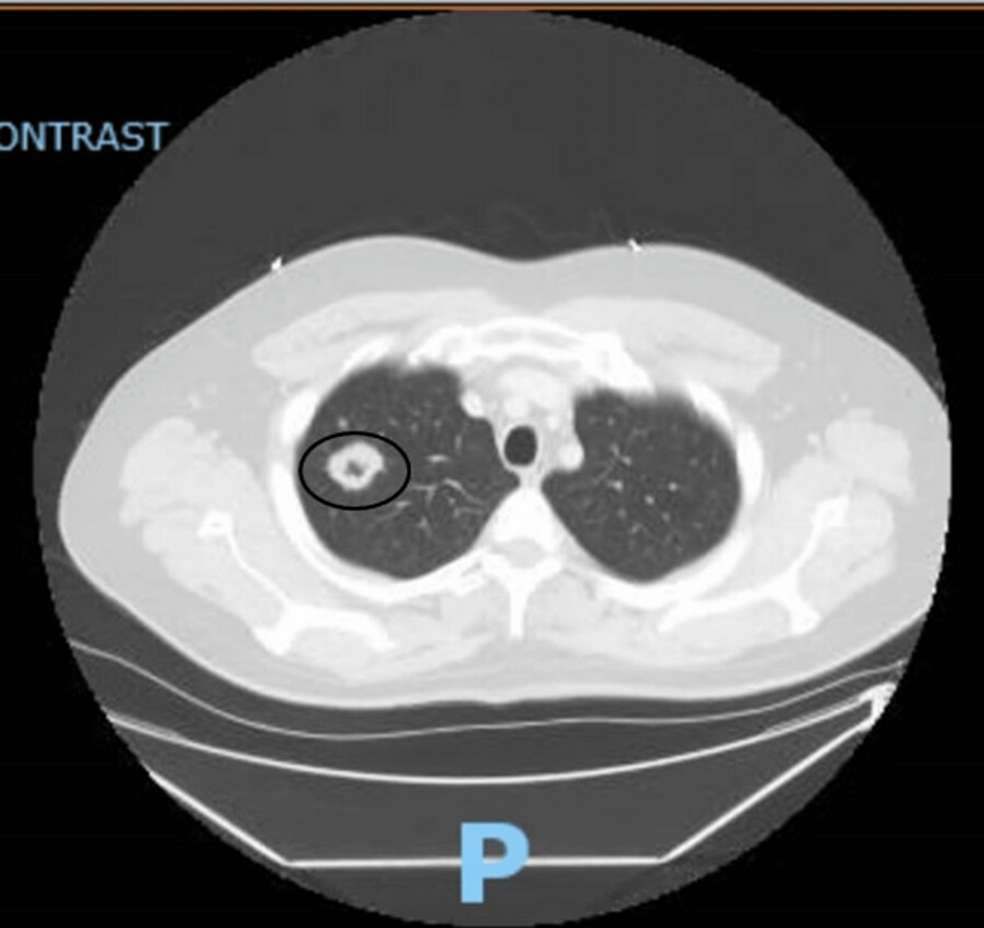
CT of the chest with a cavitary chest lesion.

**Figure 2:**
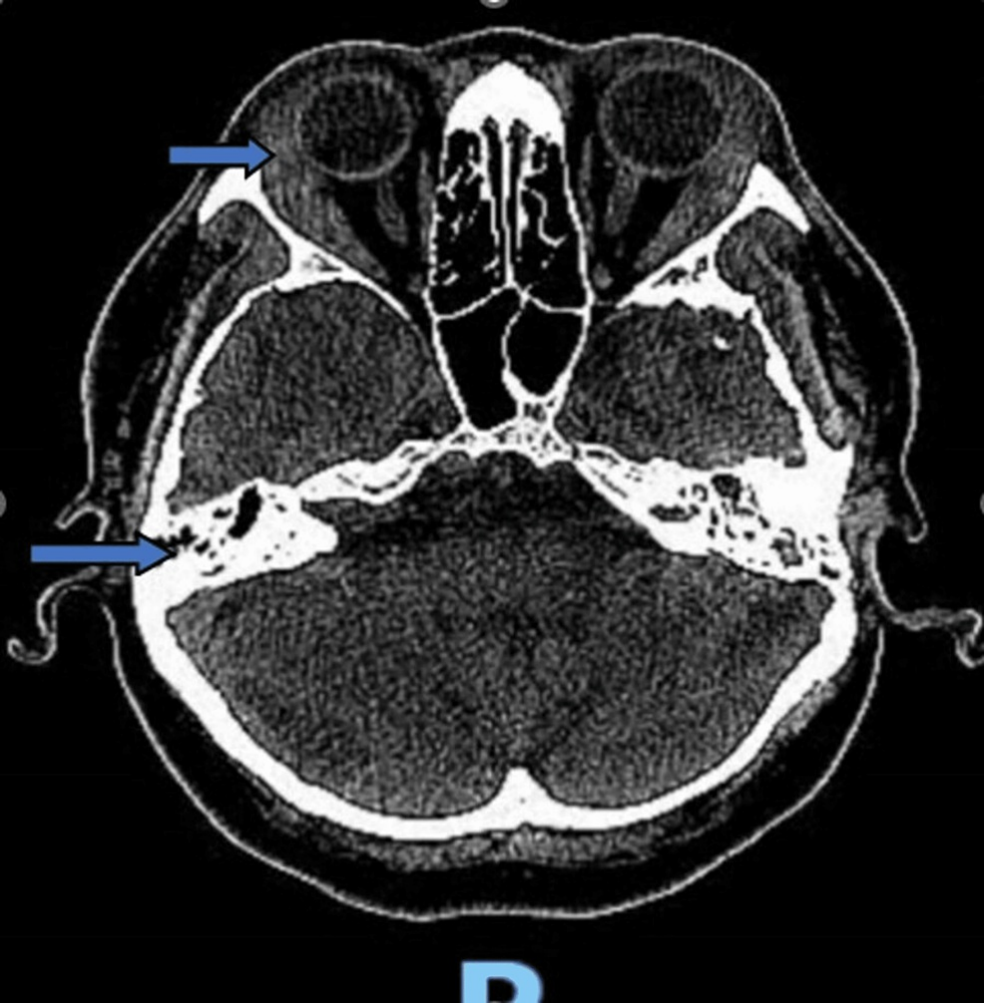
CT of the head with lacrimal hyperplasia and mastoid air cell opacification.

**Figure 3:**
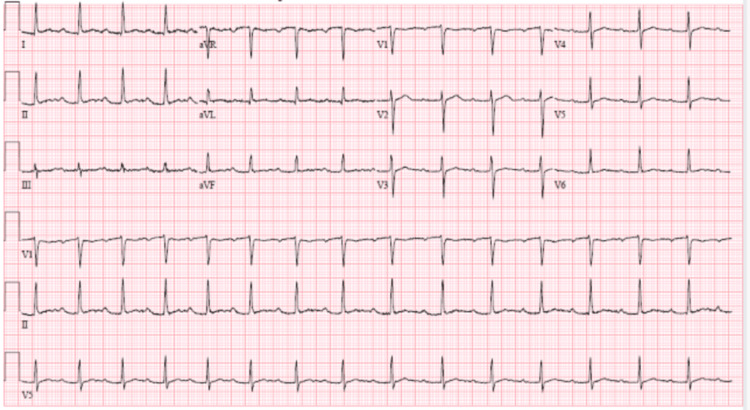
Electrocardiogram showing a first-degree atrioventricular block.

On admission, the patient was found to have a white blood cell count of 12.04 K/µL, a platelet count of 439 K/µL, an erythrocyte sedimentation rate of 98 mm/hour, a C-reactive protein of 152 mg/L, and an elevated total protein of 8.5 g/dL. His liver function tests were also abnormal, with alkaline phosphatase of 139 U/L, and alanine aminotransferase of 54 U/L. He had an initial troponin elevation of 0.12 ng/mL, and his urinalysis was positive for moderate hematuria. Initial chest X-ray (CXR) in the emergency department showed a right upper lung opacity, which was confirmed to be a 2.9 cm cavitary nodule on the chest computed tomography (CT) scan (Figure 1). CT of the head showed bilateral lacrimal gland hyperplasia and mastoid air cell opacification (Figure 2). The patient also had an electrocardiogram (ECG), which showed a first-degree atrioventricular (AV) block with a PR interval of 268 ms (Figure 3).

**Figure 4:**
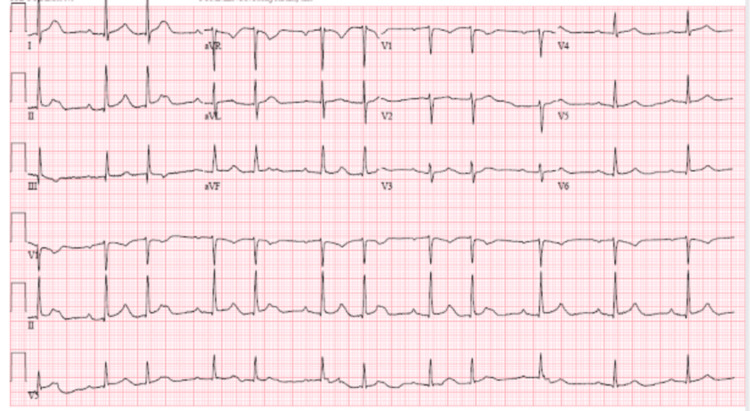
Electrocardiogram showing a second-degree Mobitz type 1 atrioventricular block.

The patient was admitted to the telemetry floors and put in an isolation room until tuberculosis was ruled out with three negative acid-fast bacilli smears. His telemetry monitoring revealed a worsening AV block that progressed to second-degree Mobtiz type 1 in the next 24 hours since baseline ECG (Figure 4). During his hospital stay, the patient complained of palpitations, and telemonitoring revealed multiple episodes of non-sustained ventricular tachycardia (NSVT). The troponin peaked at 0.17 ng/mL before trending down. Over the subsequent days, his immunology reports revealed a low positive antinuclear antigen (ANA) and rheumatoid factor (RF), but the c-ANCA was grossly positive, leading to a diagnosis of GPA.

The patient’s symptoms responded to pulse steroids intravenous methylprednisolone 1,000 mg, given intravenously for three days, followed by oral prednisone. Post-therapy telemetry monitoring did not reveal any further episodes of NSVT, and his AV block was resolved. However, he had an unrevealing echocardiogram (ECHO) and cardiac magnetic resonance imaging (MRI). The patient was started on pneumocystis pneumonia (PCP) prophylaxis and discharged with rheumatology follow-up, where he received rituximab infusions leading to complete resolution of symptoms and laboratory abnormalities.

## Discussion

GPA is a necrotizing vasculitis combining vascular wall inflammation and peri- and extravascular granulomatosis [2]. It classically involves the upper respiratory tract (92%), lungs (85%), and kidneys (77%) but may affect any system in the body [1]. Cardiac involvement, though rare, was first described by Wegener in 1939. Its prevalence (3-90%) is highly variable due to the subclinical nature of the presentation and lack of sensitive routine diagnostics to identify involvement, with a higher percentage described in severe forms and autopsy [3-5]. Many patients with lung and kidney involvement remain silent in presentation with cardiovascular symptoms and are mostly found only on postmortem analysis. However, it is crucial to consider it while managing GPA, as undiagnosed cardiac abnormalities in GPA tend to prove fatal [3,6]. The difficulty in diagnosis can be due to overlap syndromes and the need for histopathological evidence. This adds to the delay in diagnosis with a mean time from onset to diagnosis at 15 months, ranging between immediate to 15 years [7].

An essential component of diagnosing and monitoring GPA is the presence of ANCAs, with diffuse cytoplasmic fluorescence, directed against PR3 in 75% of cases and much more rarely against myeloperoxidase. c-ANCA is present in approximately 90% of systemic forms and 50% of localized forms directed against PR3 in most cases [2]. There are multiple methods and criteria to diagnose GPA. According to the American College of Rheumatology, GPA can be distinguished from other vasculitides with 88% sensitivity and 92% specificity if two or more of the following criteria are met: (i) urinary sediment containing red blood cell casts or more than five red blood cells per high-power field, (ii) abnormal findings on the chest radiograph, (iii) oral ulcers or nasal discharge, and (iv) granulomatous inflammation on biopsy [8].

Cardiac complications may present in a variety of ways. Pericarditis is the most common presentation of cardiac disease (35-50%) in the GPA population, commonly presenting as acute tamponade or chronic constriction with pleuritic chest pain and pericardial friction rubs. Other cardiac manifestations include cardiomyopathy (30%), coronary artery disease (12-50%), valvular (common in mitral and tricuspid) involvement (6-21%), and conduction abnormalities (6-17%) [3,4,7]. The presentation can sometimes mimic classic anginal chest pain secondary to coronary arteritis and myocardial ischemia [5].

It is imperative to keep Wegener’s part of the differential diagnosis in any non-specific illness with cardiac involvement, including culture-negative endocarditis, as both present with similar systemic findings (fever, malaise, weight loss, mass lesions, and vasculitis) [7,9,10]. In addition, with polar opposite treatments for both conditions, performing early immunological testing is vital to reduce the progression of the disease and further complications, including permanent cardiac, renal, and pulmonary damage [7].

A study that followed 1,829 patients diagnosed with Wegener’s over 21 years showed an increased risk of acute myocardial infarction with a hazard ratio (HR) of 2.47 and 1.64 in three months and 10 years after diagnosis, respectively [11]. The presence of heart failure was markedly elevated with an HR of 7.22 and 2.07 in three months and 10 years after GPA diagnosis, respectively [11]. Another retrospective cross-sectional analysis found that hospitalized patients with GPA had a higher prevalence of coronary artery disease and heart failure [12]. The studies concluded with a significantly high HR of cardiovascular morbidity in GPA patients at as early as three months to 10 years. This high risk prompts the need for cardiovascular surveillance in GPA, potentially benefiting preventative management and treatment strategy.

Disease involving the conduction system is seen in approximately 17% of all the cardiac cases reported, with common ones including atrial tachycardia, atrial fibrillation, and atrial flutter. Other conduction defects varying in severity can also be recognized, including intraventricular conduction defects, first- and second-degree heart blocks, and a complete heart block [5,7]. Ventricular arrhythmias are more common in patients with underlying cardiomyopathy, myocardial ischemia, or cardiac masses. They are rarely seen in patients without any structural damage [13]. Atrial arrhythmias are more common than ventricular owing to the anatomic location of the sinoatrial (SA) node, making it an easier target when epicarditis manifests, compared to the AV node, which is more distant from epicardium [5]. Patients commonly present with syncope or palpitations and are sometimes asymptomatic initially with just subtle ECG changes as initially presented by our patient. In one study, as much as 85% of the patients had ECG changes, but not all manifested symptoms and were only identified at autopsy [9].

Histopathological evidence of GPA on the heart reveals small-vessel involvement causing necrotizing vasculitis of cardiac muscle and granulomata formation affecting diffusely or focally. Valvular involvement is commonly seen in the mitral valve, followed by the tricuspid. Medium-vessel involvement is seen in coronary arteries with a range of changes from acute necrotizing to healing stages, which can lead to myocardial infarction. Conduction disorders can be secondary to granulomata of the conduction system or arteritis of supply to SA and AV nodes. The underlying cause of these conduction abnormalities is unknown but may be due to granulomatous inflammation of the conduction pathways. Cardiac findings at autopsy show granulomatous tissue within the conduction system with fibrosis and hyalinization and the absence of necrosis. Arterial involvement showed various stages of vasculitic involvement with both fibrotic and thrombotic occlusions. The histologic findings of cardiac GPA closely resemble giant-cell myocarditis, Churg-Strauss syndrome, sarcoidosis, hypersensitivity myocarditis, and myocardial infarction. Hence, a sampling error in biopsies can completely off-track the diagnosis but does not rule out GPA [3,5,7,9].

Most reports and articles have described the use of different methods of diagnosis, including ECG, cardiac enzymes, transthoracic ECHO, CT chest, cardiac MRI, and coronary angiography [4]. In a study with confirmed GPA, ECHO found 86% of the patients with heart disease, of which at least 36% attributed GPA as the cause for the findings after ruling out other explainable co-existing conditions as the cause. The same study found that almost half (42%) were identified on routine screening with an ECHO in the absence of an indication for the test. ECHO findings include global hypokinesia, regional wall motion abnormalities, mitral regurgitation, left ventricular systolic dysfunction, and pericardial effusion. A few of these patients had a reversal of the findings (acute dilated cardiomyopathy and large pericardial effusion) on immunosuppressive therapy. Routine ECHO in all patients with active GPA could be of great value as most cases can be silent [6]. Cardiac MRI can be a helpful tool that helps physicians diagnose early cardiac involvement in Wegener’s. The most common findings include a low ejection fraction, localized wall motion abnormalities, and late gadolinium enhancement [14]. However, in cases where cardiac pathology is suspected, cardiac MRI and myocardial biopsy would be beneficial to diagnose any cardiac involvement of GPA disease [15].

Due to GPA’s fulminant nature and a high number of complications, it is crucial to start prompt treatment. The mainstay of management is combination therapy with cyclophosphamide and corticosteroids, which are used to induce remission. If it is ineffective, biological treatment (rituximab) may be considered [1]. Mortality has decreased from 82% in one year before the immunosuppressive era to 25% five years post [9]. There is an increased mortality (46%) among patients with heart pathology noted on ECHO in GPA. Increased initial treatment resistance and relapse in patients with cardiac involvement, along with other factors, were noted in a few European studies [4,6]. Though there was no clear literature on treatment outcomes when the heart is involved, more studies would help validate the association, furthering our understanding of the disease and prognosis. Cardiac complications should be managed on a case-to-case basis, and specific therapies can range from short-term medication to long-term measures, such as permanent pacemakers in high-grade AV blocks [16].

## Conclusions

Severe cardiac conduction abnormalities in Wegener’s granulomatosis, although rare, can be deadly over a short time which is why we recommend that all patients hospitalized for an initial diagnosis of Wegener’s or subsequent relapse should undergo, at the very minimum, continuous cardiac monitoring and ECHO for prompt recognition and treatment of any cardiac abnormalities. Early recognition of these findings can aid in managing and improving patients’ prognoses. In addition, it furthers our understanding of the complex natural history of cardiovascular pathologies in GPA.
